# Instruments measuring emotional-social competence in preschoolers in South Africa: A review study

**DOI:** 10.4102/ajopa.v5i0.111

**Published:** 2023-01-11

**Authors:** Cebokazi N. Mtati, Erica Munnik

**Affiliations:** 1 Department of Psychology, Faculty of Community and Health Sciences, University of the Western Cape, Cape Town, South Africa

**Keywords:** assessment, emotional social competency, instruments, preschoolers, school readiness, South Africa

## Abstract

South African children still enter mainstream education with their emotional and social well-being compromised. Therefore, an awareness of and emphasis on emotional social competencies as a domain of school readiness is essential. This review aimed to identify and describe instruments measuring emotional and social competency as a domain of school readiness in preschoolers and report on their psychometric properties. The study utilised a systematic review design. Peer-reviewed articles that met the inclusion criteria were identified from six literature databases using Boolean phrases. Grey literature was also considered. The title search yielded 3872 articles. Fifty-four articles were screened based on the abstract. From among these, four articles met the minimum threshold of 80% in the appraisal phase, proceeded to data extraction and were subjected to thematic synthesis in the summation phase. The Emotional Social Screening Tool for School Readiness (E3SR), the Emotional Competence for Screening for Preschoolers (SCE), the Social Competence for Screening for Preschoolers (SCS), the Preschool Behavioural and Emotional Rating Scale (PreBERS) and the School Readiness Screening Instrument for Grade 00 (pre–grade R) were identified as instruments that measure domains of emotional social competence in preschool children. The instruments displayed good psychometric characteristics. The E3SR and the School Readiness Screening Instrument for Grade 00 were locally constructed and deemed contextually appropriate for use in the South African context. The need for locally developed, standardised, cost-effective measures to supplement assessment in the educational environment remains a focus for further research.

**Contribution:**

This review contributed to the body of knowledge related to contextually appropriate, psychometrically sound, accessible and affordable screenings available to schools and parents to assess emotional and social competencies in preschoolers in South Africa.

## Introduction

The development of emotional and social skills or competencies is imperative and plays a vital role in children’s school readiness and adjustment (Blair & Peters, 2003; Denham et al., 2014). In this article, competency is understood as the emotional and social age-appropriate behaviours that children possess and utilise effectively, resulting in emotional and social competence to enter formal schooling. It also refers to learned skills more broadly defined to include ‘the acquisition or development of specific capacities, abilities, aptitudes or competencies’ (Gilbert et al., 2004). According to Swim (2007), the attainment of social and emotional competencies is influenced by the social and cultural context in which children develop. Bustin (2007) and Mohamed (2013) acknowledge the fact that most preschoolers are unprepared to enter formal schooling because of inadequate exposure to early childhood learning opportunities and socio-economic challenges. The need to consider contextual factors that impact schools and communities to accommodate children’s unique learning needs is receiving ongoing attention (Kokkalia et al., 2019). Contextual factors such as the readiness of educational institutions to accommodate diversity, the family’s responsiveness towards children’s readiness and broader community factors such as the effect of violence or substances on the developmental trajectory of the child (Kokkalia et al., 2019; Munnik & Smith, 2019) need to be kept in mind. A recent study conducted by Wu et al. (2020), where mothers in lower socio-economic environments diagnosed with depression tend to experience challenges in their marriage and their parenting practices, which impacted negatively on their children’s abilities to establish the emotional and social skills required to establish and maintain interpersonal relationships in the early school environment, testifies to the importance of always keeping contextual factors in mind when the child is assessed for school readiness. Children from disadvantaged backgrounds may find school adjustment and learning challenging as they need to adjust to a new school environment and need to establish relationships with peers and teachers (Munnik & Smith, 2019). If they are unable to establish these relationships, they will struggle to adjust to the various demands of conventional or formal schooling (Puckett & Black, 2002). Given that many South African children enter mainstream schooling with their emotional, social, physical and intellectual well-being compromised (Laher & Cockcroft, 2013), it is of utmost importance that emphasis is placed on the development of children’s emotional and social skills. In addition to cognitive skills, emotional and social skills are identified as important in the establishment of children’s readiness to enter mainstream education.

Children who struggle with emotional regulation or management, specifically in dealing with negative emotions, may struggle to focus on learning, whereas those who have acquired adequate emotional regulation skills or manage their emotions in socially acceptable ways, are better able to easily engage in classroom activities, thereby making learning easier for them (Denham et al., 2014). Furthermore, Schultz et al. (2010) indicate that emotional regulation skills in preschoolers in turn help them to be able to facilitate social problem-solving as well as to have the ability to engage in prosocial behaviour and effective communication instead of engaging in aggressive or oppositional behaviour. Similarly, Rademacher and Koglin (2019) indicate that children who lack emotional skills have difficulty accessing competent solutions in the face of challenging situations and tasks and tend to react in oppositional or aggressive ways to solve problems, in comparison to children who have established these skills. It is clear that age-appropriate emotional and social skills remain vital for school readiness and academic success for the preschooler (Mtati, 2020).

School readiness assessments are one way to establish whether children are ready to enter mainstream schooling. As part of establishing Grade R learners’ readiness in South Africa, Foundation Phase teachers conduct continuous assessments primarily through observation, as prescribed by the Department of Basic Education (DBE, 2014). In addition, collateral from parents and other role players such as paediatricians, social workers, occupational therapists, speech therapists and psychologists may be used to gain information about the learners’ abilities. School readiness assessments are seen as an additional source of information that might be used to establish if children are ready to enter mainstream education (Laher & Cockcroft, 2013). School readiness assessment measures can be classified as either screening or diagnostic measures. Screening measures are usually cost effective, easy to use and used by multiraters to establish if further in-depth assessment is deemed necessary (Munnik, 2018). Ştefan et al. (2009) propose that screening measures provide a relatively good indication of whether a child is likely to have mastered the targeted construct or ability (Ştefan et al., 2009). In contrast, diagnostic instruments are usually used to establish a formal diagnosis to inform specific treatment plans (Foxcroft & Roodt, 2013). Diagnostic measures are usually used by trained professionals such as psychologists or psychiatrists.

Laher and Cockcroft (2013) emphasised the lack of South Africa–based literature on emotional and social competency as a domain of school readiness. In addition, Amod and Heafield (2013) argue that there is a lack of psychometrically sound locally developed school readiness assessment tools in South Africa. Munnik (2018) adds that most of the existing measures are not appropriate for use and are not able to cater to the range of children attending schools from diverse cultural and social backgrounds in the South African context. According to the literature, most of the instruments were developed more than 20 years ago, and in a post-apartheid South African setting, these assessments are out of date and inappropriate (Mohamed, 2013; Munnik et al., 2021). A few examples of South Africa–based assessments still used by practitioners to establish children’s readiness for school are the Junior South African Individual Scales (JSAIS) (Madge et al., 1985), which assesses cognitive abilities; the Griffiths Developmental Scales III (Stroud, 2016), which assesses foundations of learning, memory and social emotional development; the Aptitude Test for School Beginners (ASB), which assesses aptitudes necessary to be school ready (Human Sciences Research Council of South Africa [HSRC], 2010) and the Vinelands Adaptive Behaviour Scales, which assesses communication, daily living skills, socialisation, motor skills, and maladaptive behaviour (Roopesh, 2019). However, most of these instruments were developed abroad, with only the JSAIS and ASB being developed locally more than 20 years ago. Limited research has been conducted on the validity and reliability of all these instruments for use in a multi-cultural South Africa (Mtati, 2020).

School readiness assessment practices prioritise motor development and broader cognitive and academic abilities and competencies as a domain of school readiness (Amod & Heafield, 2013) and exclude the assessment of the emotional and social aspects of the child (Munnik & Smith, 2019). Therefore, more effective school readiness screening instruments that assess emotional social skills are important for the accurate measurement of young children’s emotional and social abilities or competencies during their preschool years (Munnik, 2018).

This review consolidated recent literature (2008–2018) on psychometric assessments that assess emotional or social competency as a domain of school readiness. The following research questions were investigated:

1. What is the methodological quality of the studies related to psychometric assessments that assess emotional and social competency as an identified area or domain of school readiness?
2. Which instruments developed locally or abroad are currently available and appropriate to assess emotional and social competence or skills as a domain of school readiness in a multicultural South African context?
3. How is emotional social competence operationalised?
4. What are the technical qualities of the identified psychometric assessments that assess emotional and social competency in school-ready children?

## Methods

### Research design

This study used a systematic review methodology and considered peer-reviewed, full-text studies that used a quantitative design, published from 2008 to 2018. The target population was preschool children between the ages of 4 and 6 years. This study expanded on the systematic review project conducted by Munnik et al. (2015).

### Search process

The present study adopted the Preferred Reporting Items for Systematic Reviews and Meta-Analyses (PRISMA) model cited in Liberati et al. (2009). Preferred Reporting Items for Systematic Reviews and Meta-Analyses recommends that systematic reviews comprise four levels of review which include identification, screening, eligibility (quality appraisal) and summation. Studies were retrieved from two core sources: database searches and grey literature. Based on their focus on psychology and education, the following databases were searched: Academic Search Complete, EBSCOhost, Education Resources Information Center (ERIC), Google Scholar, PsycARTICLES, PsycINFO, Sabinet, Sage Online and SocINDEX. Grey literature was included in the form of unpublished South African doctoral dissertations. The following search terms were used and combined in 11 Boolean phrases: ‘emotional social competency’, ‘assessment’, ‘emotional competency’, ‘social competency’, ‘school readiness instrument’, ‘preschool’, ‘emotional social intelligence’, ‘emotional social readiness’ and ‘screening instrument’ in the identification phase of filtering.

The articles that made it through the title and abstract searches were appraised by the use of the Smith Franciscus Swartbooi (SFS) Quality Appraisal Tool developed by Smith et al. (2015). Two reviewers were independently involved in the title and abstract search process with the aim of promoting and maintaining methodological rigour. A third reviewer was identified to assist with the appraisal of the extracted articles. Appraisal was done independently. After the individual appraisals, the reviewers’ scores were compared; scores that differed were discussed until consensus was reached. There were minor discrepancies noted between the scores of the reviewers initially. Most of the discrepancies were because of differences in scoring for the methodological rigour subsection of the SFS. Both reviewers read through the sections of articles related to methodological rigour, noting the reason for discrepancies. This was resolved through discussions until an agreement was reached. Finally, once the discrepancies were dealt with, only articles with a score of 80% and above, the set threshold on SFS, were accepted to proceed to the summation phase.

### Study eligibility and appraisal

The systematic review considered South African and international studies that included school readiness assessment instruments with a focus on emotional and social competency as an identified area or domain of school readiness in preschool children between the ages of 4 and 6 years. In South Africa, the preschool population is defined by the *South African Schools Act* (Republic of South Africa, 1996), specifying that children need to enter Grade 1 in the year that they are 7 years old. Peer-reviewed, full-text studies that used a quantitative design that contained the highest level of evidence from 2008 to 2018 were included. The SFS appraisal tool was used to appraise articles. It has a total of 28 questions within three sections which include purpose of the measure, methodological rigour and general considerations. Based on the overall quality of the article, each article was appraised and scored to obtain a total score (percentage) categorised as either weak (0% – 40%), moderate (41% – 60%), strong (61% – 80%) or excellent (80% – 100%). In order to be included in the current study, each article had to achieve 80% or above to ensure that only high-quality articles were used to extract relevant information in the summation phase.

### Summation

The focus of the review was descriptive, not statistical, generalisability; therefore, thematic synthesis was employed (Gough et al., 2017), which involves the integration of findings and results aiming to provide a broad description of the research phenomenon. A self-developed data-extraction table was used to extract descriptive data (type of design, methodology and outcomes) to report on the study characteristics. Thematic synthesis was employed to gather and synthesise information relating to the research aims.

### Process results

#### Identification

The *title search* yielded a result of 3872 articles via the database. During title search, 157 duplicates were identified and removed, and a total of 3663 titles were excluded from the review as they were considered inappropriate at face value.

#### Screening

Fifty-two articles were screened by *abstract* based on the inclusion criteria. Thirty-seven abstracts were excluded because of their focus on intervention as well as the age of the participants not meeting the requirements of 4–6 years old as stipulated. Policy reports, reviews and correlation studies were excluded, as well as articles that purely focused on cognitive abilities. At this stage, a decision was made to include *grey literature* in the form of unpublished South African doctoral dissertations, because insufficient South Africa–based articles were found. The dissertation by Munnik (2018) was found on Google Scholar, and the dissertation by Mohamed (2013) was found via a preliminary search on Google.

#### Eligibility

At the end of the screening stage, 15 articles and two unpublished theses were retained for quality appraisal. Of these, only two articles and the two unpublished theses were eligible for inclusion in the *final summation* based on their scores above the 80% threshold obtained on the SFS. Articles excluded lacked detail in methodological rigour and did not report on item selection, assembling of the items, development of administration instructions and gender appropriateness.

### Ethical considerations

Permission to conduct the study (reference number: HS19/6/7) was obtained from the Humanities and Social Science Research Ethics Committee of the University of the Western Cape. Ethical guidelines to conduct a systematic review included using systematic, explicit, unbiased, transparent, rigorous and reproducible methods to synthesise and integrate evidence. To ensure that reliable and valid sources of data were used in the systematic review, search databases endorsed by the University of the Western Cape were used. Permission to use the Smith Franciscus Swartbooi (SFS) appraisal tool was also obtained from the developer. The authors of the original work were appropriately cited, so that there was no violation of copyright or intellectual property.

## Summation of the review findings

### Study characteristics

The studies included (*N* = 4) represented various countries, two studies were conducted in South Africa (Mohamed, 2013; Munnik, 2018), one in Europe (Romania) (Ştefan et al., 2009) and one in America (Washington, DC) (Epstein et al., 2009). The studies provided an overview of the development of the instrument and proceeded with a detailed discussion of the technical qualities and psychometric characteristics of the instruments. Most studies used survey design as the primary data-collection method to establish the factor structures of the tests. Qualitative methods were used to report on content and face validity. Sample sizes ranged from 1471 preschool children (Epstein et al., 2009) to 310 preschool children (Ştefan et al., 2009). Urban samples were used in all studies, with the exception of Ştefan et al. (2009), who included urban and rural samples. Stratification of samples was employed in all studies including children from low, medium to high socio-economic groupings, with similar ratios for boys and girls. English versions of the protocols were used in all studies, except the SCS and SCE (Ştefan et al., 2004), where protocols were administered in Romanian or English.

### Instruments and their characteristics

The identified instruments measuring emotional and social skills in preschoolers include the Emotional Social Screening tool for School Readiness (E3SR) (Munnik, 2018), the School Readiness Screening Instrument for Grade 00 (pre–grade R) (Mohamed, 2013), the Emotional Competence Screening for Preschoolers (SCE) and Social Competency Screening for Preschoolers (SCS) (Ştefan et al., 2009) and the Preschool Behavioural and Emotional Rating Scale (PreBERS) (Epstein et al., 2009). All of the measures were developed in the years as specified above.

The PreBERS and E3SR were identified as strength-based measures, designed to assess preschoolers emotional social skills and competencies while the SCE, SCS and The School Readiness Screening Instrument for Grade 00 (pre–grade R) were identified as measures to identify developmental and academic risk in preschool children. All instruments were appropriate for use across the preschool age group, although two of these instruments focus on the age groups between 3–5 years (PreBERS) and 4–5.5 years (School Readiness Screening Instrument for Grade 00 (pre–grade R). The E3SR focuses on the age groups between 5–7 and the SCS and SCE on 5–7.5 years (The SCS and SCE also have scales for the younger age groups, 2.5–4 years, 4–5 years), thus targeting a broader age group. As the age requirement from Grade 1 is 7 years in South Africa, it can be assumed that the scales developed for the 5–7 age group might be the most appropriate scales to use to establish readiness on an emotional social level before entry to mainstream education, Grade 1.

In terms of administration, Likert scales were used in all the instruments as the preferred rating scale. There was variability across the instruments concerning the duration of administration, ranging from 10 minutes (SCS & SCE) to 15 – 20 minutes (E3SR). Likewise, the number of items varied across instruments, ranging from 42 to 57 items. The screening instruments require either parents or teachers who are familiar with the child’s skills and behavioural traits to complete the questionnaires. The School Readiness Screening Instrument for Grade 00 (pre–grade R) (Mohamed, 2013) is the only instrument of the four that has a shortened version. Shortened versions are usually easy to administer and more cost effective, and they assist with screening to establish if a more comprehensive assessment needs to be conducted (Kruyen et al., 2013).

### Theoretical and operational definitions

The instruments operationalised emotional and social competence by covering multiple subdomains, with their respective items linked to each domain. The items included in the various domains and subdomains of each instrument were closely linked to their theoretical and operational definitions. Table 1 provides an overview of the theoretical definitions as well as the domains and subdomains as operationalised in the instruments.

**TABLE 1:** Theoretical and operational definitions.

Name and theoretical definitions	Operational definitions and subdomains
**E3SR (Munnik, 2018)** **Emotional competence** Emotional competencies are directed by the child’s internal sense of self and are mostly focused inward. Emotional competencies include the subdomains of emotional maturity, emotional management, positive sense of self, mental well-being and alertness, which would enable the child to cope with age-appropriate challenges in emotion-eliciting situations across contexts. **Social competence** Having a more interpersonal or relational focus, the focus is on the relationship with the external world or environment, and thus it focuses on interactional skills including relationships with people and cooperative endeavours such as play.	**Emotional maturity:** The ability to be self-reflective about choices and actions and how they might impact self and others. **Emotional management:** The ability to become aware of one’s own and others’ emotions, to identify emotions, to understand these emotions in context and to regulate these emotions appropriately. **Independence:** The ability to initiate behaviour and take responsibility for actions in a developmentally appropriate way. **Sense of self:** The ability to hold onto a coherent and constructive sense of self that is not subject to situational outcomes. **Mental well-being and alertness:** Mental well-being: the presence of a general sense of well-being and the absence of significant symptoms that are not age-appropriate and do not fit the specific situation. Alertness: the ability to be attentive and to answer age-appropriate questions. **Social skills and confidence:** The ability to interact with others in a developmentally appropriate way. **Prosocial behaviour:** Behaviour and actions that are to the benefit of others. **Compliance with rules:** The ability to comply with and to follow rules in specific settings. **Communication skills:** The ability to use language and nonverbal expression clearly and effectively in the service of expressing thoughts, feelings and needs.
**The School Readiness Screening Instrument (Mohamed, 2013)** **Emotional domain** The ability for emotional expression, regulation and understanding, which involve perception and expression of emotion analysis and understanding of emotion and the ability to regulate emotion in self and others. **Social domain** The three components of thinking, feeling and behaviour to achieve social tasks.	**Empathy:** A social and moral emotion which involves an interaction of cognitions and affect in response to another’s emotional state. **Emotional regulation:** Processes that are used to manage and change one’s emotional state and emotion-related motivational and physiological states and how emotions are expressed behaviourally. **Self-confidence:** No definition provided. **Interpersonal competencies:** No definition provided. **Social regulation behaviour:** No definition provided. **Social graces:** Basic manners or the skills of elementary interaction.
**SCE and SCS (Ştefan et al., 2009)** **Emotional competency** Emotional competency is defined as the ability to be self-sufficient in dealing with emotion-eliciting situations in order to ensure adaptation to the social context. **Social competency** Social competency is defined as the ability to manifest socially acceptable behaviours with positive outcomes, which allow people to achieve their goals. It refers to the evaluative component of social behaviours and includes social skills or specific behaviours enacted in order to adapt to a specific social context.	**Emotion understanding**: The receptive and expressive understanding of emotions. **Emotional expressiveness**: The ability to convey emotional messages in a socially acceptable way and being able to manage emotions. **Emotion regulation:** The ability to evaluate, monitor and modify emotional reactions. **Compliance with rules**: The ability to act in accordance with rules and follow directions. **Interpersonal skills**: The ability to interact with other children and adults. **Prosocial behaviours:** A wide range of voluntary actions, directed at others’ benefit.
**PreBERS (Epstein et al., 2009)** No theoretical definitions are provided in this article. The authors refer to BERS (Behavioural and Emotional Rating Scale) for theoretical definitions.	**Emotional regulation:** The child’s ability to regulate or manage his or her behaviour in social situations with peers or adults. **School readiness:** The child’s language, preliteracy and attention-to-task skills. **Social confidence:** The child’s ability to socially engage and interact with peers. **Family involvement:** The child’s participation and relationship with his or her family.

*Source*: Extracted from Mtati, C.N. (2020). *A systematic review: Instruments that measure emotional and social competency as a domain of school readiness of preschool children in South Africa.* Maters dissertation. University of the Western Cape. Retrieved March 02, 2022, from http://hdl.handle.net/11394/7668

SCE, Emotional Competence Screening for Preschoolers; SCS, Social Competency Screening for Preschoolers; PreBERS, Preschool Behavioural and Emotional Rating Scale; E3SR, Emotional Social Screening Tool for School Readiness.

Table 1 shows that Mohamed (2013), Munnik (2018) and Ştefan et al. (2009) provided theoretical definitions of emotional and social competency as separate constructs. They divided social and emotional skills into two distinct but interrelated domains. There were similarities in the definitions, as they all viewed *emotional competency* as inclusive of the way that a child deals with and is able to cope with emotions in different contexts. For Munnik (2018), emotional competency is inward-focused behaviour that is driven by the child’s internal sense of self that allows the child to manage with age-appropriate challenges. For Mohamed (2013), emotional competency is the ability to express and understand emotions and the ability for emotional regulation in self and others. For Ştefan et al. (2009), emotional competency is related to the child’s independence in dealing with emotion-provoking situations. The authors’ definitions of *social competency* also portrayed similar understandings, being inclusive of interactions and engagement with the social environment to achieve certain goals or tasks. Munnik (2018) defined social competency as focusing on relationships with the external environment and on interactional relationships with people and cooperative activities such as play. Mohamed (2013) viewed social competency as the child’s way of thinking, feeling and behaving to achieve social tasks. Ştefan et al. (2009) describe social competency as the ability to exhibit socially acceptable behaviours with positive outcomes that allow children to achieve their goals. Epstein et al. (2009) did not include theoretical or conceptual definitions in their article, as the main focus of the article was on the establishment of the scientific standards of the PreBERS and not on the construction per se.

### Operational definitions

The most comprehensive coverage was provided by Munnik (2018), who included five subdomains of emotional competency (emotional maturity, emotional management, independence, sense of self and mental well-being and alertness) and four subdomains of social competency (social skills or confidence, prosocial behaviour, compliance with rules and communication skills). Ştefan et al. (2009) covered three subdomains of emotional competency (emotional understanding, emotional expression, emotional regulation) and three subdomains of social competency (compliance with rules, interpersonal skills, prosocial behaviour). Similarly, Mohamed (2013) covered three subdomains within the emotional domain (empathy, emotional regulation and self-confidence) and three subdomains within the social domain (interpersonal competencies, social regulation behaviour and social graces). Epstein et al. (2009) viewed emotional and social competency as one construct with four subdomains (emotional regulation, school readiness, social confidence and family involvement). Emotional regulation and social or interpersonal skills were important domains identified in all of the studies.

### Psychometric properties of the instruments

Table 2 provides a summary of the instruments’ scientific characteristics, inclusive of validity and reliability indices.

**TABLE 2:** Validity and reliability indices per instrument.

Name	Reliability	Validity
E3SR	Cronbach’s alpha analysis (over 0.95) indicated good to excellent reliability coefficients in the identified domains and subdomains of the E3SR.	**Face or content validity** established through a Delphi process with a group of 11 experts. **Construct validity:** Confirmatory factor analysis: confirmed a nine-domain structure of the E3SR, with a move towards model fit. Exploratory factor analysis: principal component analysis identified nine components, of which eight were retained. Emotional maturity, emotional management, sense of self and communication were retained while independence, mental well-being and alertness and compliance with rules were recommended for revision. **Item difficulty and analyses:** Discussed and explained in the item selection process during the construction phase. Delphi study assisted with item selection. **Item analyses:** Reported on and discussed internal consistency and reliability.
School Readiness Screening Instrument	Cronbach’s alpha analysis (over 0.7) indicated sound reliabilities for the emotional and social subdomains.	**Construct validity:** Exploratory factor analyses reduced the total pool of items for the emotional social domains to 32 items. A shortened version of the screening instrument was compiled, with 14 items in the emotional and social domains.
SCE and SCS	**Internal consistency:** (Cronbach’s alpha) was high with values over 0.80. The α values ranged between 0.80 and 0.93 for the SCE–P and SCE–E and between 0.89 and 0.95 for the SCS–P and SCS–E. **Test–retest reliability:** The test–retest coefficients for teacher and parent forms of SCE and SCS for 5–7.5 age group at a 3-month interval indicated values in the 0.72–0.83 range. **Inter-rater reliability:** Correlation coefficients significant at *p* < 0.05, and they were in the range of low agreement for both SCE and SCS.	**Face or content validity:** Evaluation by a group of eight experts. **Construct validity:** Exploratory factor analysis: item analysis was performed on the 5–7.5 cohort completed by teachers and parents. Five items from the emotional competency screening and four items from the social competency screening were dropped after analysis. **Convergent validity:** Validated against SSRS (self-controlled scale form). Pearson product–moment correlations for SCE–P and SCE–E were in the medium range. SCS from SSRS parent and teacher correlated positively with SCS–P and SCS–E in the medium to high range. **Concurrent validity:** Pearson correlations between SCS–P and SCS–E and the behaviour problem scale from the SSRS parents’ and teachers’ versions were medium negative correlations. **Predictive validity:** Screening for emotional and social competencies is a good predictor for a child’s performance on school tasks, and children’s success can be predicted by emotional and social competencies in preschool.
PreBERS	**Reliability** Alpha coefficients for the subscale and total scores were highly acceptable (above 0.83).	**Content validity:** Content analysis of several strength-based measures, including factor analysis, were reported in a previous study by Epstein et al. (2009). **Criterion validity:** *T*-test results showed significant differences between children with and without disabilities (*p* ˂ 0.001). Hedges effect sizes: moderate to large.

*Source*: Extracted from Mtati, C.N. (2020). *A systematic review: Instruments that measure emotional and social competency as a domain of school readiness of preschool children in South Africa.* Maters dissertation. University of the Western Cape. Retrieved March 02, 2022, from http://hdl.handle.net/11394/7668

SSRS, Social Skills Rating System; SCE, Emotional Competency Screening for Preschoolers; SCS, Social Competency Screening for Preschoolers; PreBERS, Preschool Behavioural and Emotional Rating Scale; P, parent version; E, Educator version; E3SR, Emotional Social Screening Tool for School Readiness.

#### Reliability

**Internal consistency:** All instruments demonstrated good to excellent internal consistency. Cronbach’s alpha analysis indicated good to excellent reliability over 0.95 in the identified domains and subdomains of the E3SR. The Cronbach’s alphas were high, with values over 0.80 for the domains in the SCE and SCS scales, and high values over 0.70 for the emotional and social domains in the School Readiness Screening Instrument for Grade 00 (pre–grade R). Cronbach’s alpha for the domains of the PreBERS was also high, with values over 0.83.

**Test–retest reliability:** Test–retest coefficients for teacher and parent forms of SCE and SCS for the 5–7.5 age group at a 3-month interval indicated values in 0.72–0.83 range. Thus, test–retest coefficients showed good stability of the scale over a 3-month interval. Test–retest reliability was not assessed and reported upon for the E3SR, PreBERS or the School Readiness Screening Instrument for Grade 00 (pre–grade R). It was mentioned as the focus for future research.

**Inter-rater reliability:** Correlation coefficients were significant at *p* < 0.05 and in the range of low agreement for both SCE and SCS. Inter-rater reliability was also not assessed and reported on in the other studies. It was recommended as a focus for future research.

#### Validity

**Face and content validity:** Ştefan et al. (2009) used experts to establish if constructs are measured similarly in the parents’ and teachers’ forms of the SCE and SCS, while Munnik (2018) used experts to establish if the items are representative of the stated domains and subdomains of the E3SR. The establishment of face and content validity for the PreBERS and Readiness Screening Instrument for Grade 00 (pre–grade R) was mentioned but not expanded upon in Epstein et al. (2009) and Mohamed (2013).

**Construct validity:** Munnik (2018) employed exploratory factor analysis that yielded an eight-factor structure and reduced the total number of items for the E3SR to 41. She also employed confirmatory factor analysis to establish a model fit, which suggested a move towards model fit. Mohamed (2013) performed exploratory factor analysis that confirmed a three-factor structure for the emotional subdomain and a four-factor structure for the social subdomain reducing the total number of items to 34. Furthermore, Mohamed (2013) also created a shortened version of the questionnaire with six items in the emotional and eight items in the social domain. Epstein et al. (2009) employed an exploratory factor analysis which yielded a four-factor structure with a total of 57 items. Ştefan et al. (2009) did not perform factor analysis.

**Convergent and concurrent validity:** The SCE and SCS were validated against the Social Skills Rating System (SSRS, self–controlled scale form). Correlations were in the medium to high range for the 5–7.5 age group on the SCE and SCS parents’ and teachers’ formats. Correlations between SCS–P (parents’ version) and SCS–E (educators’ version) and the Behaviour Problem scale from the SSRS parents’ and teachers’ versions were medium negative correlations.

**Criterion validity:** Epstein et al. (2009) concluded that the PreBERS was able to distinguish between children with and without disabilities. Ştefan et al. (2009), Epstein et al. (2009) and Munnik (2018) concluded that the PreBERS, SCS and SCE and E3SR can be used with confidence to identify children’s strengths and weaknesses in the domain of emotional and social competence as a prerequisite for entry into mainstream education. The authors also provided guidance on future directions for research such as the establishment of convergent validity, concurrent validity and further validation studies (Epstein et al., 2009; Munnik, 2018). The need for longitudinal studies was also emphasised (Ştefan et al., 2009).

In sum, the articles provided a synopsis of the construction of the instruments, their theoretical definitions and how the instruments were operationalised. They also reported on the research conducted to establish the psychometric properties of the respective instruments and the methodological criteria used to investigate reliability, factor structure and validity of the instruments.

### Implications and recommendations

The primary contribution of this review is that it assists in the identification of instruments that measure social and emotional skills as a domain of school readiness that might be applicable for use in the South African context. The review expands existing early childhood research by identifying the underlying constructs and their operationalisation in the assessment of emotional and social competence in preschoolers. This study highlighted the need for ongoing refinement of existing scales and argues for a focus on the development of more instruments to complement and aid in existing practices in the educational environment to assess emotional and social skills in preschool children. Future research should include the development of screening and diagnostic measures that focus on assessing emotional and social competencies and skills as a domain of school readiness, which are easily accessible, culturally appropriate and available for use by educators, parents and professionals such as psychologists in the South African context.

## Conclusion

There is a lack of screening and diagnostic measures currently available to assess emotional and social skills as an area or domain of school readiness in preschoolers in South Africa. The perception that many developed assessment tools are not effective and undervalue the emotional and social competencies as part of school readiness assessment is still the dominant perception. The screening and school readiness assessment measures available abroad are not standardised for the South African population and therefore not appropriate for use within a multicultural South African context. More effective school readiness screening instruments that assess emotional and social skills are important for the accurate screening of young children’s emotional and social competencies during the preschool years. This review highlighted the need for ongoing engagement in research pertaining to children’s emotional and social skills as an important area or domain of school readiness. The need for appropriate diagnostic instruments is also highlighted as a means to identify learners in need of further intervention.
